# The Role of NLRP3 Inflammasome in Lupus Nephritis

**DOI:** 10.3390/ijms222212476

**Published:** 2021-11-19

**Authors:** Camila Barbosa Oliveira, Camilla Albertina Dantas Lima, Gisele Vajgel, Paula Sandrin-Garcia

**Affiliations:** 1 Division of Nephrology, Clinical Hospital, Federal University of Pernambuco, Recife 50670-901, Brazil; camilabarbosalyra@hotmail.com (C.B.O.); giselevajgel@hotmail.com (G.V.); 2 Laboratory of Immunopathology Keizo Asami (LIKA), Federal University of Pernambuco, Recife 50670-901, Brazil; camilladelima@gmail.com; 3 Department of Oceanography, Federal University of Pernambuco, Recife 50670-901, Brazil; 4 Department of Genetics, Federal University of Pernambuco, Recife 50670-901, Brazil

**Keywords:** inflammasome, NLRP3, lupus nephritis, systemic lupus erythematosus

## Abstract

Lupus nephritis (LN) is the most frequent and severe of systemic lupus erythematosus (SLE) clinical manifestations and contributes to the increase of morbidity and mortality of patients due to chronic kidney disease. The NLRP3 (NLR pyrin domain containing 3) is a member of the NLR (NOD-like receptors), and its activation results in the production of pro-inflammatory cytokines, which can contribute to the pathogenesis of LN. In this review manuscript, we approach the relation between the NLRP3 inflammasome, SLE, and LN, highlighting the influence of genetic susceptibility of NLRP3 polymorphisms in the disease; the main functional studies using cellular and animal models of NLRP3 activation; and finally, some mechanisms of NLRP3 inhibition for the development of possible therapeutic drugs for LN.

## 1. Introduction

Systemic lupus erythematosus (SLE) is a chronic autoimmune inflammatory disease characterized by a broad spectrum of clinical manifestation and prognosis. Lupus nephritis (LN) is the kidney disease of SLE, and it is one of the most frequent and severe complications [1,2]. The prevalence of LN varies depending on the studied population, with higher rates among African-Americans (69%) and Hispanics (61%) compared to Caucasians (29%) [3]. In addition to the high prevalence, LN is associated with significant morbidity and mortality. The Systemic Lupus International Collaborating Clinics (SLICC) group evaluated 1.827 multi-ethnic patients for nearly five years and concluded that LN was associated with higher risks of end-stage renal disease (ESRD) (hazard ratio (HR) = 44.7, 95% CI: 6.1–329.7; *p* < 0.001) and death (HR = 3.2, 95% CI: 1.6–6.5; *p* = 0.002) [1].

The pathogenesis of SLE is characterized by the interaction of genetic susceptibility, environment, infection, immunity, and hormone levels, resulting in loss of tolerance and sustained autoantibody production. Immune dysfunction represents a critical feature in the development of SLE, with focus on the adaptive immune system. The abnormal activation and function of T cells and B cells results in the generation of high levels of autoantibodies, circulating immune complexes, and inflammatory cytokines, which leads to tissues and organs inflammation and damage by immune complex deposition [4].

More recently, interest in the role of the innate immune system in the pathogenesis of SLE has increased dramatically. Monocytes, macrophages, and dendritic cells are innate immune cells and represent the first line of defense against pathogens. These cells have pattern recognition receptors (PRRs), which recognize pathogen-associated molecular patterns (PAMPs) and damage-associated molecular patterns (DAMPs). Nucleotide-binding leucine-rich repeat receptors, or NOD-like receptors (NLRs), are cytosolic PRR activated by PAMPs and DAMPs [5]. The NLR pyrin domain containing 3 (NLRP3) is a subtype of NLR and has been extensively studied in autoimmune diseases. The interaction between NLRP3, the adaptor protein apoptosis-associated speck-like protein containing a caspase recruitment domain (ASC), and pro-caspase-1 forms a protein complex described as the NLRP3 inflammasome.

The activation of NLRP3 inflammasome occurs through a two-signal mechanism. The first signal is provided by the activation of Toll-like receptors (TLRs), interleukin-1 (IL-1) receptors, or tumor necrosis factor receptors (TNFRs), which stimulate the transcription factor NF-κB to produce pro-forms of IL-1β and IL-18. The second signal is triggered by PAMPs or DAMPs, promoting the oligomerization of NLRP3 with ASC, leading to the proteolytic cleavage of pro-caspase-1 into caspase-1. The activated caspase-1 cleaves pro-interleukin-1β and pro-interleukin-18 into active pro-inflammatory cytokines, interleukin-1β (IL-1β), and interleukin-18 (IL-18) [5,6]. Additionally, caspase-1 also cleaves gasdermin-D into its active form: a protein with the ability to form transmembrane pores, leading to cell lysis and the release of cytoplasmic IL-1β and IL-18, which is a process called pyroptois [7].

In SLE patients, the increased rate of apoptosis combined with an inefficient clearance of apoptotic cells leads to an accumulation of apoptotic cell debris [8,9,10]. Persistent apoptotic cells debris may progress to secondary necrosis with the accumulation of DAMPs and activation of inflammasome NLRP3. Several DAMPs can activate NLRP3 inflammasome including reactive oxygen species, extracellular ATP, nucleic acids, and extracellular matrix components [11,12,13,14]. Nucleic acids represent the main self-antigens in SLE. Immune complexes formed after antibody recognition of DNA and RNA antigens can act as DAMPs and activate inflammasome NLRP3 via Toll-like receptors (TLR) activation [15,16].

Neutrophil extracellular traps (NETs) are structures composed of granule proteins bound to chromatin fibers and produced by activated neutrophils to kill extracellular pathogens [17]. NETs are effective activators of NLRP3 inflammasome in human and murine macrophages, and SLE patients are more likely to activate the inflammasome in response to NETs than healthy controls [18]. Low-density granulocytes (LDGs) are a distinct subset of pro-inflammatory neutrophils detected in patients with SLE. LDGs have an increased capacity to form NETs and an upregulated expression of proteins and enzymes of NETs formation, leading to a higher exposure of immunostimulatory molecules and a greater ability to activate inflammasome [17]. In addition, experimental studies suggest a reduced NETs clearance in SLE patients, resulting in more inflammasome NLRP3 activation, greater release of inflammatory cytokines, and significant tissue damage [19,20]. The IL-18 released by inflammasome NLRP3 activation induces NETosis, leading to a feed-forward loop of NETs formation and inflammasome activation [18].

Another process associated to SLE susceptibility is an abnormal autophagy. In physiological conditions, autophagy is a natural regulatory mechanism involved in the removal of unnecessary or dysfunctional cellular components and in the control of inflammasome activation [21]. SLE patients can present with a blockade of macrophage autophagy, with hyperactivation of inflammasome probably caused by the accumulation DAMPs and loss of degradation of inflammasome proteins and IL-1β [22].

The NLRP3 inflammasome has been implicated in the pathogenesis and progression of LN and other kidney diseases. Renal mononuclear cells (dendritic cells and macrophages) and some non-immune renal parenchymal cells (endothelial cells, parietal epithelial cells, tubular epithelial cells, and podocytes) express the components of inflammasome NLRP3 and can activate the inflammasome in response to various PAMPs and DAMPs [23]. Ischemic reperfusion kidney injury can lead to cell damage and death with release of endogenous DAMPs, activation of NLRP3 inflammasome, and renal tubular epithelial cell necrosis [24]. The damaged muscles in rhabdomyolysis release catalytic iron and myoglobin heme, inducing oxidative stress in renal tubular epithelial cells, activation of NLRP3 inflammasome, and acute kidney injury [25]. Other kidney diseases such as diabetic nephropathy, obstructive nephropathy, and crystal nephropathy can also activate the inflammasome NLRP3, induce inflammation, and lead to a progressive glomerular and/or tubulointerstitial fibrosis [26,27,28].

In LN, most kidney injuries are a consequence of the intense inflammatory response, and the NLRP3 inflammasome has been frequently implicated in the pathogenesis of tissue damage. Consequently, the inhibition of the NLRP3 inflammasome could be a beneficial strategy for the treatment of LN. Therefore, this review will discuss recent evidence of genetic susceptibility of NLRP3 polymorphism in SLE and LN patients, functional studies of NLRP3 activation in cellular and animal models, and possible therapeutic inhibitors of the NLRP3 inflammasome in LN.

## 2. Genetic Susceptibility of *NLRP3* and SLE

The NLRP3 gene, also known as NALP3 or cryopyrin, was initially studied for Hoffman et al., when the researchers were mapping the gene responsible for hereditary periodic fever syndromes [29]. NLRP3 is located in chromosome region 1q43-q44 and contains 3 kb upstream of the transcription start site (exons and introns) and 2 kb downstream of the stop codon (37,953 kb total) [30]. About 60 Single Nucleotide Polymorphisms (SNPs) are present on the NLRP3 gene and the five most studied in SLE are rs10754558, rs4612666, rs3806268, rs35829419, and rs4352135, due to Minor Frequency Allele (MAF) and/or possible functional impact. However, two SNPs are more associated with SLE: rs35829419 (C > A) is a missense variant with gain-of-function with overexpression of IL-1β and IL-18 and rs10754558 (C > G) located in 3′UTR gene region, in which the C allele decreases the stability of mRNA in relation to the G allele and consequently IL-1β and IL-18 cytokines secretion [31,32]. In fact, these SNPs are already associated with various autoimmune and inflammatory diseases; however, in SLE, only a few studies verified the rs35829419 and rs10754558 association with disease and/or its clinical manifestations.

In one of the first studies, Pontillo et al., verified the association between 14 SNPs in seven inflammasome genes, among them rs35829419 and rs10754558, in 144 SLE patients and 158 healthy individuals from Southern Brazil. No significant association was observed between the studied NLRP3 SNPs and susceptibility to SLE or clinical manifestations. Nevertheless, the authors observed a significant association between the rs2670660 G allele and an increased risk for SLE (OR = 2.06, *p* value = 1.50^−5^) and LN when compared patients with and without manifestation (OR = 2.09, *p* value = 4.0^−4^), suggesting a predisposing effect of this allele for LN [33]. This study showed, for the first time, the influence of variants in inflammasome genes to SLE development and LN.

Lee and Bae performed a meta-analysis of the 30 studies that evaluated the rs35829419 and rs10754558 polymorphisms in autoimmune and inflammatory diseases. A total of 8069 patients and 8824 controls were included in the meta-analysis. The meta-analysis showed no association between NLRP3 rs35829419 SNP and autoimmune or inflammatory diseases [34]. Considering the results observed specifically for SLE in this meta-analysis, a significant association of the rs10754558 C allele with SLE (OR = 1.465, *p* = 0.002), but not to the rs35829419 C allele, was observed in two studies by Pontillo et al. [33,35]. NLRP3 rs10754558 SNP also had a significant susceptibility to the autoimmune and inflammatory diseases in the Latin American population [34].

In the Chinese population, the SNPs rs3806268, rs4612666, and rs10754558 were evaluated in 400 SLE patients and 400 healthy controls. No association was observed between SNPs and SLE susceptibility. In addition, the authors evaluated the association among three NLRP3 SNPs and SLE activity, serum cytokines, complements, and autoantibodies levels. The rs4612666 TT genotype was associated with an increased risk for SLE progression in codominant and recessive models, respectively (OR = 2.08, *p* value = 0.02; OR = 2.34, *p* value = 0.01). Therefore, an increased risk of SLE disease activity was observed also for the haplotype GTG (in the order of rs3806268, rs4612666, and rs10754558) (OR = 2.08, *p* value = 0.02). The authors also observed that levels of IL-1β and IFN-g were significantly increased in the rs3806268 AA and rs10754558 CC genotype SLE patients, respectively. Likewise, the rs3806268 GG was associated with low C4 and the rs4612666 TT genotype was associated with anti-SSA positive rate in rs10754558 CG genotype carriers [36]. A previous study had been performed in 127 Chinese SLE patients evaluating another SNP in NLRP3 (rs4353135); however, no distinct frequency was observed between patients and healthy individuals. Interestingly, an association was observed in SNP rs2043211 (CARD8) with increased risk of SLE in males when compared to female patients (OR = 2.11, *p* value = 0.017) [37].

Recently, Cruz et al., evaluated the association of inflammasome gene SNPs in 132 SLE patients and 154 healthy controls in the Northeast Brazilian population. The rs10754558 was initially associated with SLE development; however, when adjusted for Bonferroni correction, it did not reach the statistical significance (*p* = 0.012 and padj = 0.074). It is interesting that when the authors performed the association with SLE clinical manifestations, the rs10754558 G allele was significantly associated with LN (OR = 3.88, *p* = 0.0004) when comparing patients with or without kidney involvement, according to a dominant model [38]. In the Iranian population, three NLRP3 SNPs were evaluated, two variants of gain (rs10754558 and rs4612666) and one of loss function (SNP rs6672995). The variant rs6672995 located in the regulatory region of NLRP3 was previously associated with a decrease of IL-1β production [39]. When SLE patients and healthy controls were compared, only rs10754558 was associated with an increased risk to SLE development (G allele OR = 1.97, *p* value ≤ 0.001; GG genotype OR = 2.82, *p* = 0.003 and combined genotypes GC + GG OR = 2.02, *p* value = 0.016). Interestingly, when SLE patients were stratified according to clinical and laboratorial parameters, all SNPs tested were statistically associated. For laboratorial parameters, C and G alleles for rs4612666 and rs10754558 were associated with lower age onset, respectively. In addition, patients with the C allele for rs461266 presented higher concentration for C-reactive protein (CRP), erythrocyte sedimentation rate, anti-double stranded DNA (anti-dsDNA) antibodies, and C3 and C4 complement. In the same away, patients with the G allele for rs10754558 presented increased levels of CRP and anti-dsDNA antibodies but lower levels of C3 and C4. These results showed the involvement of these variants in disease activity and its possible application to serum level markers. Finally, the authors observed an association of neurological manifestations with SNPs rs4612666 and rs10754558, and three SNPs for kidney involvement [40].

Finally, it is very interesting to note the association of the NLRP3 inflammasome with clinical and laboratory features in SLE patients, specially related to LN activity and severity, which is a clinical condition strongly associated to a high morbidity and mortality among patients with lupus.

## 3. Functional Studies of *NLRP3* in Cellular and Animal Models

Although association studies have suggested polymorphisms and pathways that are NLRP3-related, functional studies are requested for validation of these data. There are four main mouse models of LN available, termed NZB, (NZB × NZW) F1 hybrid (NZB/W), MRL/lpr, and BXSB strains, which differ among developmental LN, susceptibility to renal lesions, and time to present albuminuria and proteinuria [41,42]. Although the murine models seem to show similar immunological mechanisms to human LN in experimental studies, LN is a complex disease, and animal models have limitations and cannot exactly simulate human disease expression or predict response in humans. Consequently, immune and renal cells have been increasingly used in LN studies [43,44,45].

The relationship between high NLRP3 levels and tissue damage in LN was already recognized in previous studies suggesting that an inhibition of NLRP3 inflammasome activity can diminish the acute inflammation and kidney damage associated in mice and human models [46,47,48,49,50]. NLRP3/ASC/caspase 1 inflammasome acts mainly activating NF-κB by mydssome and the TRAF6/IRAK complex, increasing IL-1β and IL-18 levels and enhancing Th17 cell polarization [23,48,51,52]. Additionally, other pathways can activate NLRP3-inflammasome inducing a positive feedback mechanism [23]. Necrosis and apoptosis may promote Ros formation by the RIP3 (receptor interacting protein kinase 3) complex, which promotes damage of the plasma membrane and increases NLRP3 inflammasome activation [53]. In the same way, ATP from sites of tissue injury and inflammation may activate P2X7, which is an immune cell receptor that is able to promote NLRP3-inflamassome activation [54,55,56].

In a renal environment, NLRP3-inflammasome inhibition has been related to reduced IL-1β and IL-18 levels, circulating anti-dsDNA antibodies, albuminuria, and kidney lesions, leading to a less severe LN [51,53,57]. Fu et al. showed an activation of the NLRP3 inflammasome and expression of IL-1β in both LN class IV and class V from human kidney biopsies, while they were not detected in the podocytes of normal kidneys, suggesting a NLRP3 participation in LN pathogenesis [57].

**Figure 1:**
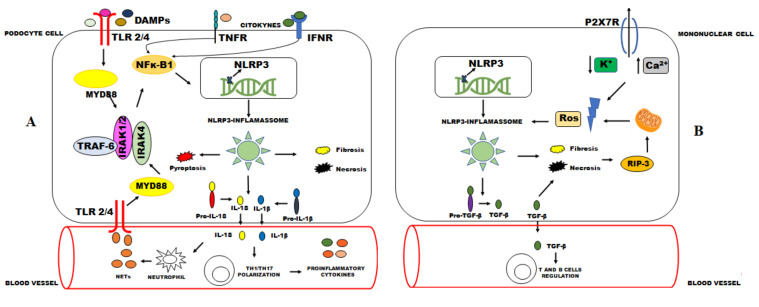
Possible mechanisms of NLRP3-inflammasome activation in podocytes and mononuclear phagocytes (**A**) and mononuclear phagocytes (**B**). (**A**): DAMPs and pro-inflammatory cytokines can activate the NLRP3 inflammasome via the NFκ-B signaling pathway. The increasing of NLRP3 transcriptions by NFκ-B induces the NLRP3-inflammasome activation. The NLRP3 inflammasome cleaves pro-IL-1β and pro-IL-18 by caspase 1. These cytokines may cause kidney damage through an induction of inflammation and polarization of Th17 cells. IL-18 can also induce the formation of NETs by neutrophils, increasing stimuli by DAMPS. (**B**): A second signal provided by K^+^ efflux and Ca^2+^ flux mediated by P2X7 receptor induces Ros formation, enabling NLRP3-inflammasome activation. Similarly, necrosis and fibrosis promoted by NLRP3-inflammasome activation induce Ros formation through RIP3. These mechanisms together with the formation of TGF-β appear to contribute to kidney damage in LN. NLRP3: NLR pyrin domain-containing-3; TLR: Toll-like receptor; Myd88: Myeloid differentiation factor 88; TRAF-6: TNF receptor-associated factor 6; DAMPs: Damage-associated molecular patterns; IRAK-1: Interleukin 1 receptor-associated kinase 1; IRAK-4: Interleukin 1 receptor-associated kinase 4; NFκ-B: Nuclear factor kappa B; IL: Interleukin; Th: T helper; NETs: Neutrophil extracellular traps; K^+^: Potassium ions; Ca^2+^: Calcium ions; P2X7R: P2X7.

It is important to emphasize that NLRP3 inflammasome seems to have complex effects in kidney disease and LN establishment. Kidney injury without IL-1β and IL-18 participation and non-increased levels of NLRP3 have suggested that non-canonical functions of NLRP3 such as tumor growth factor (TGF)-beta signaling, epithelial–mesenchymal transition, necrosis, and fibrosis may promote the severity of kidney injury [23,53,58,59]. Additionally, previous studies showed NLRP3 deficiency as a protective factor against post-ischemic AKI, from the NLRP3 pathway not related to inflammasome signaling and IL-1β or IL-18 action [24,60]. Surprisingly, NLRP3 and ASC suppression contributed to LN establishment in a study performed by Lech et al., inducing massive expansion of T and B cells, increase of dendritic cell and macrophage activation, and an increased expression of pro-inflammatory mediators in autoimmunity mouse models. The immuno-regulatory role of NLRP3 and ASC would be related to impaired TGF-βR signaling, since it is an important negative regulator of the immune system [52]. These findings show that NLRP3-inflammasoma pathways and their interactions need to be elucidated. Many possible mechanisms of action are summarized in Figure 1.

Although these other pathways seem to influence inflammatory processes, the NLRP3 inflammasome, IL-1β, and IL-18 levels are still the main targets used to identify potential molecules inhibitors in LN and kidney diseases [51,61,62].

## 4. Therapeutic Inhibitors of *NLRP3* Inflammasome in Lupus Nephritis

As previously mentioned, studies in animal models and in humans have shown that the NLRP3 inflammasome is hyperactivated in PBMCs and in the kidney tissue (podocytes and tubular cells) of SLE individuals [16,57,63,64]. Therefore, blocking this pathway seems a promising target to control LN activity. In contrast, other studies showed that NLRP3 inflammasome expression is decreased in the PBMCs of SLE patients and is negatively correlated with disease severity [52,65,66]. Thus, a deficiency of NLRP3 resulted in hyperactive immune cells with an increased expression of numerous pro-inflammatory cytokines. This may be explained by differences in expression among the cell types, study models, patients, or due to the inhibitory effect of overactivated T cells from adaptive immunity as well as crosstalk between type I IFN, TGF-β, and the NLRP3 inflammasome signaling.

Inhibitors of the NLRP3 inflammasome can act directly by blocking the protein function or indirectly by targeting P2X7 receptor, ASC, caspase-1, IL-1, or RIP3. Among direct inhibitors of NLRP3, MCC950, a selective NLRP3 inhibitor, has been shown to minimize IL-1β production in vivo and to attenuate the severity of experimental autoimmune encephalomyelitis, crystal-induced kidney fibrosis in mice, steroid-resistant asthma in an animal model, and diabetic kidney injury in mice [27,67,68,69]. In addition, it was able to reduce the expression of NLRP3, caspase-1, and IL-1 β production in the kidney of NZM2328 mice. The animals presented with less proteinuria three weeks after the treatment and had attenuated foot process effacement and lower activity scores in the kidney histology [57].

Additionally acting as an NLRP3 direct inhibitor, Tris dibenzylideneacetone dipalladium (DBA), a small-molecule palladium complex, has been shown to inhibit cell growth and proliferation in B-cell malignancies such as lymphocytic leukemia [70]. It is known that B cells mediate LN; therefore, Wu et al. tested Tris DBA in an LN-prone mouse model. Tris DBA-treated mice showed markedly reduced albuminuria, lower anti-dsDNA antibodies levels, and better kidney function compared with control mice, which also developed markedly intrinsic cell proliferation, cellular crescents, neutrophil infiltration and fibrinoid necrosis in the glomeruli, tubulointerstitial inflammation, increased activity score, and higher glomerular deposition of IgG, IgM, and C3 seen on immunofluorescence. Kidney inflammation was significantly reduced in the Tris DBA mice through regulation of autophagy mediated by the NLRP3 inflammasome, which was less expressed in the kidney tissue of treated mice. The implied mechanism seems to be the regulation of T-cell function along with phosphorylation inhibition of JNK, ERK, and p38 MAPK signaling pathways via ROS-mediated inflammation [71].

Bay 11-7082 seems to have an inhibitory effect on the NLRP3 inflammasome independent of NF-κβ, being an inhibitor of IκBα phosphorylation and the subsequent NF-κβ activation [72,73]. Zhao et al. demonstrated that NLRP3 and ASC expressions were significantly reduced after treatment with Bay 11-7082 compared to placebo-treated mice that had decreased proteinuria, blood urea nitrogen, resulting in dramatically attenuated kidney damage. Bay 11-7082-treated mice had decreased serum anti-dsDNA antibodies levels, IL-1β, less TNF-α and chemokine (C-C Motif) ligand 2 (CCL2) levels, and reduced infiltration of macrophages in the kidney tissue, resulting in a lower mortality for treated mice [47].

In addition, acting in the way of the NLRP3 and NF-κβ by indirect inhibition, epigallocatechin-3-gallate (EGCG), the major bioactive polyphenol present in green tea, has reported antioxidants and ROS scavenging properties. An inhibition of NF-κβ-mediated inflammatory responses has been shown in cancer cells exposed to EGCG [74]. One study showed that EGCG was able to prevent the development of LN in NZB/WF1 lupus-prone mice due to inactivation of the Nrf2 antioxidant pathway, which resulted in renal NLRP3 inflammasome inactivation with less amounts of IL-1β and IL-18 in kidney lysates. Caspase-1 activation was also seen in placebo-treated kidneys, with consequent p20 subunit expression, but it was significantly inhibited by EGCG treatment [46].

Another indirect way of action is the ROS inhibitor, Citral (3,7-dimethyl-2,6-octadienal), which is a major active compound in a Chinese herbal medicine Litsea cubeba, and M1, an active metabolite of ginsenoside, the major bioactive compound of ginseng, seems to be effective in a lipopolysaccharide (LPS)-induced NZB/Wf1 mice model by inhibition of NLRP3 inflammasome activation, ROS, and COX-2 production as well as Nrf2 activation [62,75]. M1 has shown significant inhibition of NLRP3 inflammasome activation in podocytes through autophagy induction [62]. In addition, M1 can also downregulate the NF-κβ-mediated signaling pathway and inhibit pro-inflammatory cytokine production in rat arthritis, tumor growth, and chronic colitis [62,76,77,78,79].

An impairment of ROS production with the use of chemical scavengers of ROS, pharmacological inhibitors of NAD(P)H oxidase, or siRNA-mediated knockdown of NAD(P)H oxidase has been shown to inhibit NLRP3 inflammasome activation in response to several stimuli [80,81,82]. Mitochondrial uncouplers are able to reduce ROS production. In this regard, niclosamide ethanolamine salt (NEN), a mild mitochondrial uncoupler used as an anthelmintic drug, has been tested. In addition to uncoupling oxidative phosphorylation with superoxide formation and mitochondrial biogenesis, NEN also seems to participate in the modulation of Wnt/β-catenin, mTORC1, STAT3, NF-κB, and Notch signaling pathways [83]. Previous data pointed out that NEN might improve diabetic kidney nephropathy [84]. ROS generation is usually accompanied by K^+^ efflux; it is not well known, but it seems possible that low intracellular K^+^ concentration enhances ROS production, which activates NLRP3 inflammasome activation independent of the channel activity of P2X7 [82]. In this manner, a recent study showed that NEN reduces glomerular inflammation with less hypercellularity and podocytes loss. Treated mice also had lower tubular injury markers such as urinary NGAL and Kim-1 with lower renal mRNA expression levels of MCP-1, IL-1β, suggesting that NEN has inhibitory effects on inflammation [85]. However, the underlying mechanisms require further studies.

Still using an indirect way, the P2X7 receptor is expressed in membranes of cells in high extracellular ATP milieu, which is usually due to pathological conditions. When activated by ATP or NETs, the P2X7 receptor increases the influx of cations such as calcium that will activate necroptosis through NLRP3 inflammasome [86,87]. There is an indirect inhibition of NLRP3 inflammasome via P2X7 and NF-kβ signaling inhibition by Brilliant blue G (BBG) attenuated LN in MRL/lpr mice. BBG treatment reduced the serum levels of IL-1β and IL-17 and the Th17/Treg cell ratio. These results were also obtained by the specific siRNA silencing of P2X7 in vivo. The results of BBG treatment in reducing LN inflammation were confirmed in NZM2328 mice with AdIFNα-accelerated disease [51]. In kidney tissue, RIP3 activation leads to necroptosis and NLRP3 inflammasome activation in podocytes. A recent study using MRL/lpr mice tested GSK872, an inhibitor of RIP3, MLKL, and caspase-1, which reduced glomerular and tubulointerstitial inflammation with less IgG and C3 deposition in the kidney tissue [53].

Data regarding the PIM kinase family and its inflammatory-mediated cytokines response have been attracting attention. The oncogene PIM-1 is a serine threonine–protein kinase that activates NFAT-1 and NLRP3. The inhibition of PIM-1 has shown attenuation of inflammation in colitis and allergen-induced airway responses in mice models [88,89]. Recently, Fu et al. showed a reduction of kidney NLRP3 and caspase-1 activation with less IL-1β and proteinuria when AZD 1208 was used, which is an inhibitor of PIM-1 in NZb/WF1 mice compared to placebo. PIM-1 inhibitors reduced the calcium influx, which downregulated NLRP3 inflammasome activation. The authors also tested a selective PIM-1 inhibitor (SMI-4a) on MRL/lpr mice, resulting in attenuation of proteinuria onset and significantly prolonged survival of treated mice [73].

In summary, direct and indirect NLRP3 inhibitors are future possibilities in LN treatment; however, studies evaluating its therapeutic effects are rare and limited to animal models. Another future perspective in the treatment of inflammatory diseases is gene editing. A recent study of Xu et al. evaluated the use of CRISPR/Cas9 (a third-generation gene editing tool) to directly disrupt the key molecule, NLRP3, at the genomic level of macrophages and treat multiple inflammatory diseases. The NLRP3 knockout inhibited the NLRP3 inflammasome activation in vitro and in vivo and seemed to be a promising therapy for NLRP3-dependent inflammatory diseases [90].

## 5. Conclusions and Future Directions

LN is a frequent and severe clinical manifestation in SLE with a multifactorial pathogenesis as well as significant morbidity and mortality. Activation of the NLRP3 inflammasome has been extensively evaluated as a contributing factor in the pathogenesis and progression of LN. This review manuscript discussed the influence of NLRP3 inflammasome polymorphisms in LN, with studies showing a significantly higher risk of LN in patients with NLRP3 genetic variants, and the inflammasome NLRP3 activation in animals and cellular models, including the activation in kidney tissue and urine samples of LN patients. Furthermore, the review evaluated the consequences of NLRP3 inflammasome inhibitors in LN animal models.

NLRP3 inflammasome seems to be a promising therapeutic target for LN, and a wide range of direct and indirect inhibitors are being evaluated to block this pathway and control LN activity. However, the evidence is limited to a few studies in animal models. Therefore, future work can contribute, providing a more detailed understanding of the regulatory mechanisms of the NLRP3 inflammasome in kidney cells of patients with LN, and clinical trials are needed to evaluate the effects of NLRP3 inflammasome inhibitors in preventing the progression of lupus kidney disease. In addition, more gene editing studies are needed to evaluate the effect of NLRP3 knockout in specific diseases such as SLE and LN.

## Data Availability

Not applicable.
